# Cholangioscope-assisted endoscopic retrograde appendicitis therapy for the treatment of appendicitis complicated with fecal stones: a case report

**DOI:** 10.1055/a-2325-2233

**Published:** 2024-06-05

**Authors:** Qi Zhao, Ruixia Wang, Maofeng Sun, Shulei Zhao

**Affiliations:** 1 34708 Shandong Provincial Hospital Affiliated to Shandong First Medical University, Jinan, China; 2 518873 Shandong University, Shandong Provincial Hospital, Jinan, China; 3 518873 Shandong First Medical University, Jinan, China

Acute appendicitis typically occurs when the opening of the appendix is blocked, most commonly by an impacted fecalith 1 . Surgical interventions for acute appendicitis often come with the drawbacks of increased postoperative complications and a high rate of removing the healthy appendix 2 . In 2012, a minimally invasive technique known as endoscopic retrograde appendicitis therapy (ERAT) was proposed. This method involves flushing out fecal stones and eliminating the blockage through the natural passage 3 . However, ERAT relies on X-ray and involves a complex procedure 4 . Drawing inspiration from the successful application of ultraslim endoscopes in treating complex bile duct stones, we incorporated a 9-Fr cholangioscope to assist with ERAT in managing appendicitis complicated with fecal stones. In this report, we present a case wherein this innovative approach was used.

A 15-year-old girl presented with recurrent right lower abdominal pain, leading to a diagnosis of acute appendicitis with fecal stones as revealed by ultrasound. Considering the patient's young age and her expressed wish to preserve her appendix, a decision was made to proceed with cholangioscope-assisted ERAT for treatment ( Video 1 ).

This video demonstrates the process of cholangioscope-assisted endoscopic retrograde appendicitis therapy for the treatment of appendicitis complicated with fecal stones.Video 1

A standard colonoscope with a 3.7-mm biopsy channel was used to reach the ileocecal region, where it was observed that the opening of the appendix appeared normal. Subsequently, a cholangioscope was inserted through the biopsy channel and successfully entered the appendix cavity ( Fig. 1 ). This allowed for the visualization of a significant amount of yellow fecal stones. With direct visual guidance from the cholangioscope, the fecal stones were effectively flushed out of the appendix cavity ( Fig. 2 ). After multiple flushing attempts ( Fig. 3 ), it was observed that the mucosa of the appendix cavity had become smooth and the lumen was no longer obstructed ( Fig. 4 ). The colonoscope and cholangioscope were then withdrawn. The pattern diagram of the treatment process for the appendicitis complicated with fecal stones in this case is shown below ( Fig. 5 ). Following the surgery, the patient experienced significant relief from the symptoms and signs. During a follow-up phone call 1 month later, the patient reported no abdominal pain.

**Fig. 1:**
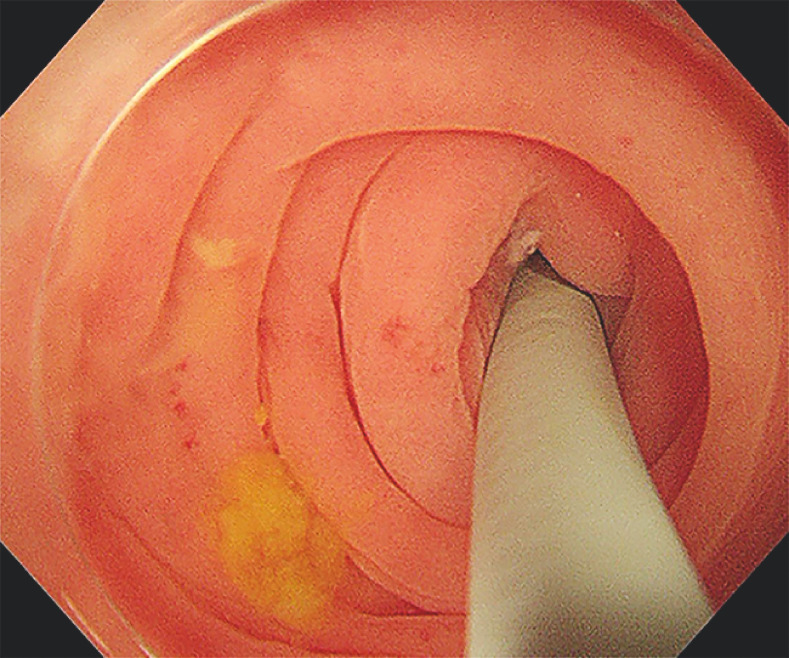
Insertion of the cholangioscope into the opening of the appendix as observed during colonoscopy.

**Fig. 2:**
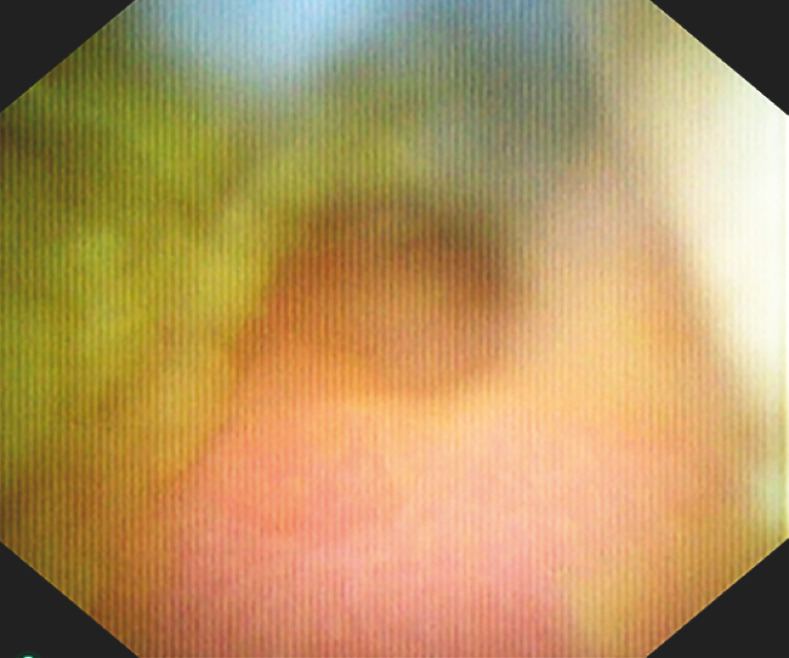
With direct visual guidance from the cholangioscope, the fecal stones were effectively flushed out of the appendix cavity.

**Fig. 3:**
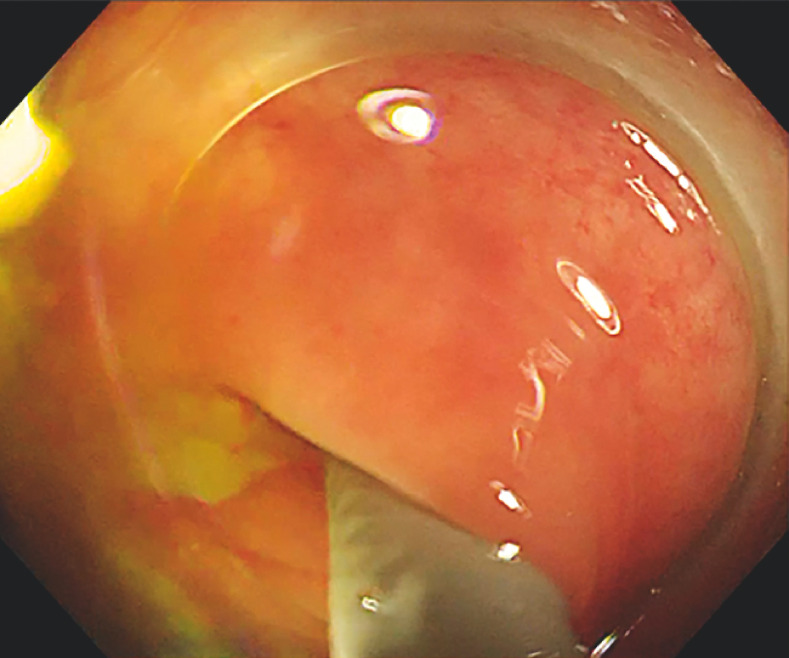
After multiple flushing attempts, the fecal stones were flushed out of the appendix cavity under the colonoscope.

**Fig. 4:**
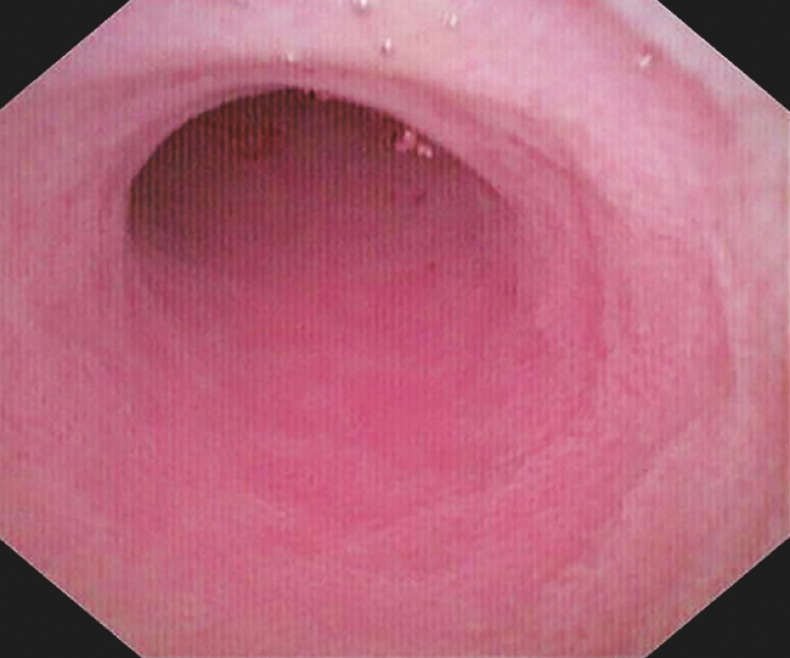
It was observed that the mucosa of the appendix cavity had become smooth and the lumen was no longer obstructed under the cholangioscope.

**Fig. 5:**
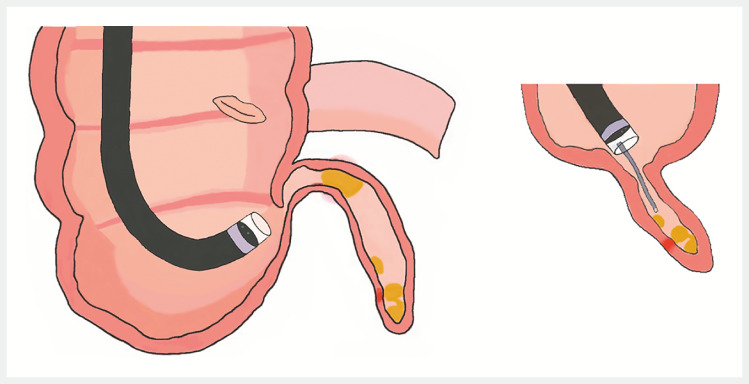
The pattern diagram of the treatment process for the appendicitis complicated with fecal stones in this case.

Compared to traditional ERAT, this operation does not rely on X-ray and provides a more comprehensive treatment for appendiceal fecal stones. The effectiveness and safety of ultraslim endoscope-assisted ERAT have been confirmed.

Endoscopy_UCTN_Code_TTT_1AQ_2AF

## Notes

Endoscopy E-Videos https://eref.thieme.de/e-videos
*E-Videos* is an open access online section of the journal *Endoscopy* , reporting on interesting cases and new techniques in gastroenterological endoscopy. All papers include a high-quality video and are published with a Creative Commons CC-BY license. Endoscopy E-Videos qualify for HINARI discounts and waivers and eligibility is automatically checked during the submission process. We grant 100% waivers to articles whose corresponding authors are based in Group A countries and 50% waivers to those who are based in Group B countries as classified by Research4Life (see: https://www.research4life.org/access/eligibility/ ). This section has its own submission website at https://mc.manuscriptcentral.com/e-videos .
